# Gene Sequences of Potential Targets of Insecticidal PF2 Lectin Identified from the Larval De Novo Transcriptome of the Mexican Bean Weevil (*Zabrotes Subfasciatus*; Boheman 1833)

**DOI:** 10.3390/insects11110736

**Published:** 2020-10-27

**Authors:** Irlanda Lagarda-Diaz, Miguel Ángel Hernández-Oñate, José Ángel Huerta-Ocampo, Ana M. Guzmán-Partida, Joy Winzerling, Dawn Geiser, Luz Vázquez-Moreno

**Affiliations:** 1 CONACYT-Universidad de Sonora, Hermosillo, Sonora 83000, Mexico; irlanda.lagarda@unison.mx; 2 CONACYT-Centro de Investigación en Alimentación y Desarrollo, A.C., Hermosillo, Sonora 83304, Mexico; miguel.hernandez@ciad.mx (M.Á.H.-O.); jose.huerta@ciad.mx (J.Á.H.-O.); 3 eCentro de Investigación en Alimentación y Desarrollo, A.C., Hermosillo, Sonora 83304, Mexico; gupa@ciad.mx; 4 Department of Nutritional Sciences, Division of Agriculture, Life and Veterinary Sciences, University of Arizona, Tucson, AZ 85721, USA; jwinzerl@ag.arizona.edu (J.W.); dlgeiser@email.arizona.edu (D.G.)

**Keywords:** coleopteran insect, pest insect, RNA-seq, Transcriptomics

## Abstract

**Simple Summary:**

The Mexican bean weevil *Zabrotes subfasciatus* is a major insect pest of stored beans. We have previously reported that the PF2 lectin, which is a protein found in the desert wild legume *Olneya tesota* (Palo Fierro), is toxic to *Z. subfasciatus* by inhibiting its early larval development. The use of proteomic means allowed us to identify PF2 targets in the midgut of *Z. subfasciatus* larvae. However, efforts to completely elucidate the insecticidal mechanism of PF2, as well as novel potential targets for insecticidal compounds, have been hindered by the lack of available genomic and proteomic information of non-model insects. Therefore, in this work we massively sequenced and analyzed the transcripts expressed in the larval stage of *Z. subfasciatus*, which is the first transcriptome reported for this insect. A total of 29,029 transcript sequences were identified, of which 30 sequences encode putative targets of PF2. The functional characteristics and biochemical, biological, or molecular roles for 15,124 sequences were established by means of bioinformatics tools. This study significantly increased the available genetic resources for *Zabrotes* and related insect species and will be helpful for any kind of future study that requires information on genes or protein sequences.

**Abstract:**

The available genomic and proteomic information of non-model organisms is often underrepresented in public databases hindering their study at molecular, cellular, and physiological levels. Information on *Zabrotes subfasciatus* (Mexican bean weevil) is poorly represented in databases, yet it is a major pest of common beans. We report the transcriptome of *Z. subfasciatus* larvae; transcripts were sequenced using an Illumina RNA-Seq technology and assembled de novo identifying 29,029 unigenes with an average size of 1168 bp and an N50 value of 2196 bp. About 15,124 unigenes (52%) were functionally annotated and categorized. Further analysis revealed 30 unigene sequences encoding putative targets of the insecticidal PF2 lectin. The complete deduced amino acid sequences of eight selected proteins potentially related to insecticidal mechanism of Palo Fierro 2 (PF2) were used for predicting probable N-glycosylation sites and analyzing phylogenetic relationships with insect sequences. This work provides a dramatic increase in the genetic resources available for Coleopterans and set the basis for developing future studies on biological aspects and potential control strategies for *Z. subfasciatus*.

## 1. Introduction

The Mexican bean weevil (*Zabrotes subfasciatus*; Boheman 1833) is the main postharvest pest of the common bean (*Phaseolus vulgaris* L.) in Mexico. This bruchid (Coleoptera: Bruchidae) is native to Central and South America and is found in tropical and subtropical regions of Africa, Southeast Asia, India, and parts of Europe [1]. The common bean is a legume of critical agricultural importance in Mexico, Central and South America, and is an essential component of the diet in these regions. It is also a staple food source, rich in protein, vitamins, minerals, and fiber [2].

Infestation of stored beans with *Z. subfasciatus* decreases viability and nutritional value of the beans and causes considerable financial losses (25–40%) for producers [3]. Intensive application of broad-spectrum insecticides can significantly reduce weevil populations to control this pest, however such treatments also raise consumers’ concerns about residual chemical toxicity and potential negative effects of the insecticides on human health, as well as associated environmental pollution [4]. Alternatives that replace synthetic insecticides to control this pest with harmless options include the use of entomopathogenic microorganisms, as well as essential plant oils that are toxic to *Z. subfasciatus* [4,5]. Several plant proteins are entomotoxic (e.g., lectins, ribosome-inactivating proteins, inhibitors of proteolytic enzymes, glycohydrolases, arcelins, chitinases, canatoxin and modified forms of storage proteins [6]. Some of these non-synthetic alternatives are highly selective for specific pests, which reduces the impact on non-target species, including mammals. 

Lectins are proteins/glycoproteins of non-immune origin, that exhibit reversible and highly-specific binding to simple or complex carbohydrates. They are abundant in legume seeds and have demonstrated antimicrobial, antitumor, and insecticidal properties, and they have been successfully engineered into a variety of crops as a pest management strategy [7,8]. Interaction of legume lectins with different glycoproteins or glycan structures in the insect gut disrupts nutrient absorption and assimilation, and interferes with several physiological processes that consequently results in growth retardation or death [8].

Palo Fierro 2 (PF2), a lectin from the desert wild legume *Olneya tesota* (desert ironwood) is highly toxic to the first larval stages of *Z. subfasciatus*. PF2 selectively recognizes complex triantennary tetrasialylated carbohydrates and binds structures in the midgut of *Z. subfasciatus* larvae [9]. We have hypothesized that the insecticidal mechanism involves PF2 interaction with glycoproteins essential for larval development [10], e.g., PF2 recognizes larval α-amylase and alters its biological function, however this cannot fully explain the insecticidal activity of PF2 because of the coexistence of non-glycosylated isoforms of larval amylases [11]. In addition, several potential targets of PF2 in the midgut of *Z. subfasciatus* larvae have been identified, including V-type proton ATPase, prohibitin, ATP-synthase subunit alpha, mitochondrial-processing peptidase, a growth factor-like glycoprotein, arginine kinase, polyubiquitin, actin, ATP-dependent RNA helicase, α-tubulin, odorant receptor, cytochrome c oxidase, mitochondrial, sensorial, structural, and defense proteins, and proteins involved in the electron transport chain [12,13].

Elucidation of the role played by most of the glycoproteins interacting with PF2 in the midgut of *Z. subfasciatus* is limited due to the scarce number of protein and nucleotide sequences (26 and 40, respectively) available in public databases for *Z. subfasciatus* and taxonomically-related organisms. Hence, we sought to use Illumina RNA-Seq technology to assemble the larval transcriptome, and to characterize its genes, rather than relying on a reference genome and/or the study of the expression of its glycoproteome. We report the first transcriptome analysis of *Z. subfasciatus* larvae and we use the obtained database to identify putative candidate unigene sequences as potential targets for PF2 lectin. This information will pave the way to gain insights into the insecticide mechanism mediated by the PF2 lectin. This, in turn, will allow the design of alternate pest control strategies and will facilitate our understanding of essential developmental processes in *Zabrotes* and taxonomically related insects.

## 2. Materials and Methods

### 2.1. Insect Culture

Colonies of *Z. subfasciatus*, kindly donated by the Entomology Laboratory of Universidad of Sonora, were raised for several generations on *Phaseolus vulgaris* ´Peruano´. Insects were handled under controlled conditions (27 °C, 65–75% relative humidity, and light for 12 h daily) as previously reported [14].

### 2.2. Larvae Sampling and RNA Isolation

Total RNA was isolated from whole larvae of 12, 13 and 14 days-old using the RNeasy Mini Kit (Qiagen Inc., Valencia, CA, USA). RNA samples were assessed for quality with an Advanced Analytics Fragment Analyzer (Agilent Technologies, Santa Clara, CA, USA) using the High Sensitivity RNA Analysis Kit (Agilent Technologies, Santa Clara, CA, USA), while quantity was estimated with the Quant-iT RiboGreen RNA Assay Kit (Thermo Scientific, San Jose, CA, USA) according to the manufacturer’s instructions.

### 2.3. cDNA Library Preparation and Illumina Sequencing

Given satisfactory quality and quantity, RNA was prepared for library builds using the TruSeq RNA Sample Preparation Kit v2 (lllumina, San Diego, CA, USA). When the library build was completed, sample quality and average fragment size were assessed with the Advanced Analytics Fragment Analyzer using the High Sensitivity NGS Fragment Kit (Agilent Technologies, Santa Clara, CA, USA). Quantity was assessed with a Kapa Library Quantification kit for Illumina NGS (Kapa Biosystems, Boston, MA, USA). After final library quality control was completed, equimolar sample was pooled and clustered for sequencing on the HiSeq2500 system (Illumina, San Diego, CA, USA). The sequencing run was performed using Illumina Rapid-Run SBS chemistry (TruSeq Rapid SBS Kit, Illumina, San Diego, CA, USA) in paired-end mode with a read length of 100 bp paired-end reads (2 × 100 paired-end) bp. The raw reads were deposited in the National Center for Biotechnology Information (NCBI) Sequence Read Archive (SRA) under the accession number PRJNA646957.

### 2.4. RNA-Seq Data Processing and De Novo Assembly

Data processing and analysis was done in collaboration with the University of Arizona Genetics Core (Tucson, AZ, USA). RNA-seq reads from the Illumina HiSeq2500 platform were trimmed to remove adapter sequences and filter out low quality reads, the bases with quality lower than 3 were trimmed from the trailing ends of reads. Trimming was done with trimmomatic [15], specifying a minimum sequence length of 20. After filtering, a total of 92 million paired reads were used to complete the de novo transcriptome assembly. To reduce the data set to a manageable size for assembling transcripts, digital normalization was done using the khmer tool [16] with the trimming parameters suggested in the khmer online protocol for mRNASeq assembly [17]. After completing the digital normalization steps at a coverage level of 50X, about 11 million paired-end reads and 1.7 million single reads remained. These reads were assembled using SOAPdenovoTrans [18] with khmer settings ranging from 19 to 89. The assembly selected for further analysis was generated with khmer = 19, due to its relatively high N50/N90 contig statistics and because lower khmers tend to be more favorable when trying to assemble lower abundance transcripts. Assembled transcripts with a length less than 200 bp were removed. The statistics and quality of the assembly were determined with the Benchmarking Universal Single-Copy Orthologs (BUSCO) v3 tool [19] using the Insecta odb9 database (n = 1658) as reference.

### 2.5. Unigene Annotation and Classification

The functional annotation of assembled unigenes was performed comparing the sequences with those of protein databases including NCBI non-redundant (NR, https://www.ncbi.nlm.nih.gov/), RefSeq (https://www.ncbi.nlm.nih.gov/refseq/) and SwissProt (https://www.uniprot.org/) using blastx (E-value < 1^−10^ for NCBI nr and an E-value < 1 × 10^−5^ for the other databases). Additionally, we compared the assembled unigenes of *Z. subfasciatus* with the predicted proteins of phylogenetically-related organisms including, *Anoplophora glabripennis* (Asian long-horned beetle; [20]), *Dendroctonus ponderosae* (Mountain pine beetle; [21]), *Leptinotarsa decemlineata* (Colorado potato beetle; [22]) and *Tribolium castaneum* (Red flour beetle; [23]) using blastx with an E-value < 1 × 10^−5^. In all blast searches, the best hits were chosen to annotate the unigene sequences, and in the case of inconsistencies, annotation was prioritized in the following order: NR (non-redundant), SwissProt, RefSeq. We also used InterPro databases to identify the protein domains. Functional categorization of unigenes based on Gene Ontology (GO) and Kyoto Encyclopedia of Genes and Genomes (KEGG) were carried out using the BLAST2GO suite [24] using the GO-Slim mode to eliminate redundancy of GO terms and the KEGG Automatic Annotation Server (KAAS, https://www.genome.jp/kegg/kaas/) using the single-directional best hit mode.

### 2.6. Identification and Phylogenetic Analysis of Potential Targets of PF2 Lectin

According to the functional annotation, we identified the putative sequences coding for amylase, imaginal disc growth factor 4 precursor, mitochondrial-processing peptidase subunit beta, NADH-ubiquinone oxidoreductase subunit mitochondrial, prohibitin, and V-type proton ATPase. The unigene sequences selected were translated using the Open Reading Frame Finder tool of NCBI (https://www.ncbi.nlm.nih.gov/orffinder/). We searched the characteristic functional domains of these proteins and made pairwise alignments with proteins of known annotation from related species. The resulting annotations were manually verified and sequences with inconsistencies were discarded.

Unigene sequences identified for each protein were aligned using the Clustal Omega tool; redundant sequences or those with high homology (>95% identity) were eliminated from the analysis to avoid identical transcripts.

Using the deduced amino acid sequences from each identified unigene, we predicted putative N-glycosylation sites (NetNGlyc 1.0 Server at http://www.cbs.dtu.dk/services/NetNGlyc/) and analyzed their phylogenetic relationships to similar proteins of related organisms. A blastp analysis (E-value < 1^−10^, identity >30% and Query coverage >50%) was performed to find homologous sequences from other insects using as query the amino acid sequences deduced from assembled unigenes. Phylogenetic trees for each identified protein were constructed using the MEGAX software (Sudhir Kumar, AZ, USA) and the neighbor-joining method with bootstrap of 1000 replications.

## 3. Results

### 3.1. Sequence Analysis and De Novo Assembly

The sequences from *Z. subfasciatus* larval total RNA yielded a total of 92 million paired-end reads with an average length of 100 bp. Digital normalization [16], resulted in about 11 million paired-end reads and 1.7 million single reads. These reads were used to perform the de novo assembly of the *Z. subfasciatus* transcriptome [18].

**Figure 1:**
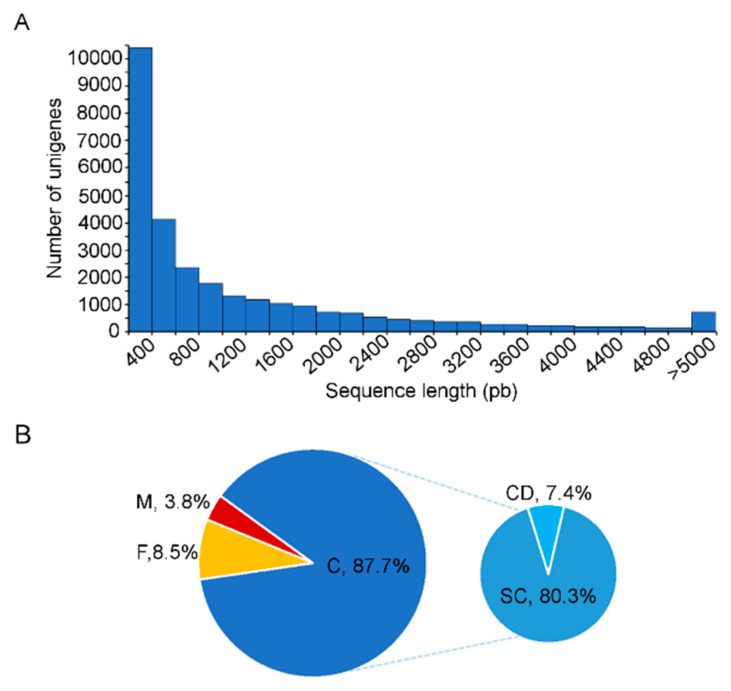
Quality analysis of de novo transcriptome assembly. (**A**). Length distribution of assembled unigenes. (**B**). Analysis of transcriptome completeness using Benchmarking Universal Single-Copy Orthologs (BUSCO) software. Pie charts show the percentage of the 1658 single-copy Insecta ortholog genes database (OrthoDB v9) covered by the assembly: C, complete genes found; SC, complete genes found in single-copy; CD, complete genes found in duplicate; F, fragmented genes found; M, missing ortholog genes.

Assembly generated a total of 29,029 transcripts (unigenes) with an average size of 1168 bp and an N50 value of 2196 bp that represented an assembled transcriptome of 33.9 Mbp with a length range of sequences from 200 to 15,454 bp (Table 1). The length distribution of assembled sequences showed that most of the unigenes were 200 and 800 bp; however, about 10,300 unigenes were more than 1000 bp in length (Figure 1A) suggesting they could correspond to full length transcripts. Further, transcriptome analysis using the BUSCO v3 tool with the Insecta database (n = 1658 BUSCO orthologs genes) indicated that 87.7% (1454) were complete; of these 80.3% (1332) were complete single-copy and 7.4% (122) were duplicates, 8.5% (141) were fragmented and only 3.8% (63) were missing (Figure 1B). A high BUSCO score is associated with a high-quality transcriptome assembly.

### 3.2. Unigene Annotation

**Figure 2:**
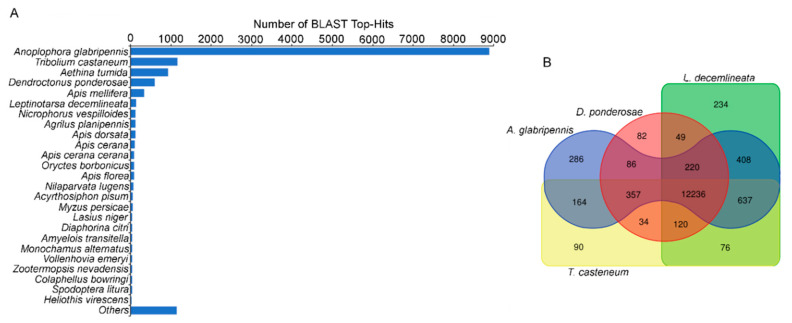
Homology analysis of the assembled unigenes. (**A**). BLAST Top-hits species taxonomic distribution. The graph shows the number of unigenes with BLASTX-Hits to the non-redundant (NR) NCBI database (E-value < 1^−10^). (**B**). Edwards-Venn diagram shows the distribution of shared and unique BLASTX-Hits of the assembled unigenes of *Z. subfasciatus* compared with related species (E-value < 1^−5^).

To identify the functions of the assembled unigenes, we compared the sequences with publicly available protein databases including, non-redundant (NR) NCBI, RefSeq and SwissProt. About 52% (15,124) of the translated sequences of 29,029 unigenes matched at least one predicted protein. The greatest number of database hits were found in RefSeq, 51.9% (15,079 unigenes), followed by NR NCBI 48.2% (14,003 unigenes) and SwissProt 36.7% (10,643 unigenes) (Table S1). The BLAST Top-hits species analysis (Figure 2A) shows the greatest number of matches is with the predicted proteins of *A. glabripennis* (63.5%), followed by *T. castaneum* (8.3%), *Aethina tumida* (small hive beetle; 6.6%) and *D. ponderosae* (4.3%). Further comparative analyses using blastx tool (E-value < 1 × 10^−5^) with proteins of the *A. glabripennis* (Ag), *D. ponderosae* (Dp), *L. decemlineata* (Ld) and *T. castaneum* (Tc) genomes shows that the greatest number of putative orthologs of *Z. subfasciatus* were obtained from the comparison with Ag (14,394 unigenes) and Ld (13,980 unigenes). A total of 12,236 sequences (42.1%) from *Zabrotes* shared putative proteins with the four insect species analyzed, while some unigenes matched only with Ag (286), Ld (234), Tc (90) or Dp, (82) (Figure 2B). Overall, 52% of the unigenes (15,079) found a match in these protein databases, however 13,950 unigenes (48%) had no hits in the blastx searches with the four species analyzed. This suggests that these sequences could represent non-conserved regions or unique proteins of *Z. subfasciatus*.

### 3.3. Protein Domains Annotation

To complete the functional annotation, we identified conserved domains using the InterPro database. Our analysis showed that 15,830 unigenes (54.5%, Table S1) were annotated and classified in 4640 InterPro families and 2748 InterPro domains; a list of the 30 InterPro domains with the greatest representation is shown in Table 2. Zinc finger C2H2-type (IPR013087; 440 unigenes), protein kinase domain (IPR000719; 195). Major Facilitator Superfamily domain (IPR020846; 137), WD40-repeat-containing domain (IPR017986; 135), and RNA recognition motif domain (IPR000504; 132) showed the greatest representation (Table 2).

### 3.4. Functional Categorization and Metabolic Pathway Annotation

**Figure 3:**
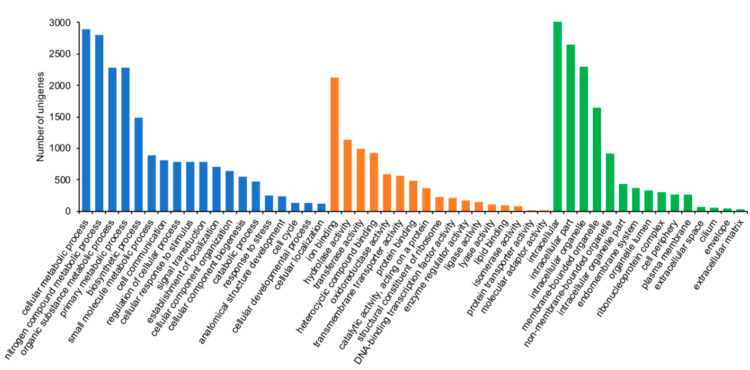
Gene Ontology (GO) classifications of the unigenes from the *Z. subfasciatus* transcriptome. Histogram shows the GO functional categories most represented based on level 3 analysis. Biological Process (blue bars), Molecular Functions (orange bars) and Cellular Component (green bars).

In order to obtain the functional categorization of the transcriptome, we compared the unigenes with the GO database using Blast2GO; the GO terms identified were mapped to obtain the GO-slim terms. A total of 11,741 unigenes (40.4%, Table S1) were annotated to at least one GO-slim term, and these were classified by the GO functional categories (Biological Process, Molecular Functions, and Cellular Component). 8743 unigenes were assigned to 39 molecular functions, 5649 unigenes to 66 biological process and 5163 unigenes to 29 cellular components; GO terms with less than 5 sequences assigned were discarded (File S1). Subsequently, the levels for GO terms based on the annotation coverage and specificity were analyzed; level 3 provided us the best specificity for greatest coverage. Level 3 GO terms distribution analysis revealed that most represented biological processes were: cellular metabolic process, nitrogen compound metabolic process, organic substance metabolic process, primary metabolic process, and biosynthetic process. The most represented molecular functions were ion binding, hydrolase activity, transferase activity, heterocyclic compound binding and oxidoreductase activity. While the top five cellular components identified were: intracellular, intracellular part, intracellular organelle, membrane-bounded organelle, and non-membrane-bounded organelle part (Figure 3).

**Figure 4:**
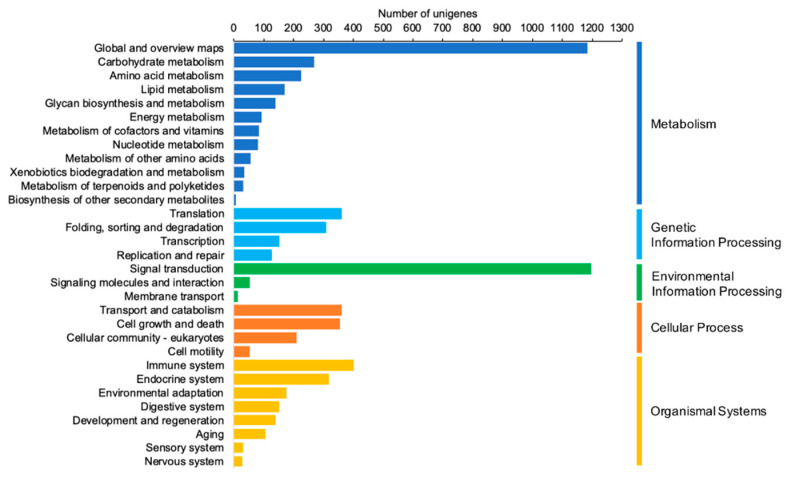
Distribution of pathways associated with the unigenes from the *Z. subfasciatus* transcriptome compared with the Kyoto Encyclopedia of Genes and Genomes (KEGG) database.

The identification of biological pathways represented in the *Z. subfasciatus* transcriptome was performed using the KEGG database. All the unigene sequences were compared with those of the KEGG database using the KEGG KAAS. This analysis allowed us to annotate a total of 8195 unigenes (28.2%) into five KEGG categories including: metabolism, genetic information processing, environmental information processing, cellular process and organismal systems. Distribution analysis showed that the most represented category was metabolism, where 268 unigenes were assigned to the carbohydrate metabolism pathway, 227 to amino acid metabolism, 169 to lipid metabolism, 140 to glycan biosynthesis and metabolism, and 92 to energy metabolism among others (Figure 4). In addition, as shown in Figure 4, a large number of unigenes were assigned to signal transduction (1195 unigenes), immune system (402), translation (361), transport and catabolism (360), cell growth and death (356), endocrine system (317), folding, sorting and degradation (310), environmental adaptation (176) and digestive system (151).

### 3.5. Identification and Phylogenetic Analysis of Potential PF2 Lectin Target Sequences

We previously reported that PF2 lectin is toxic to *Z. subfasciatus* larvae, and we identified several proteins from the midgut of *Z. subfasciatus* larvae that were specifically recognized by PF2 and thus could serve as potential targets for PF2 lectin insecticidal activity [11,12]. All the possible unigenes associated to these previously suggested PF2 targets were manually curated from the *Z. subfasciatus* transcriptome, which allowed us to identify a total of 30 different unigenes encoding putative PF2 lectin targets; of these, we were able to identify the complete open reading frame (ORF) for 8 sequences. Imaginal Disc Growth Factor 4 (IDGF4-chitinase), ATP synthase, and prohibitin were among the most represented protein families. Furthermore, generally, lectins interact with other proteins by binding to carbohydrates found on their target proteins. We analyzed the sequences of the most promising targets for PF2 for the identification of putative glycosylation sites. Our results showed that several proteins from those we analyzed had at least one putative glycosylation site (Table 3).

**Figure 5:**
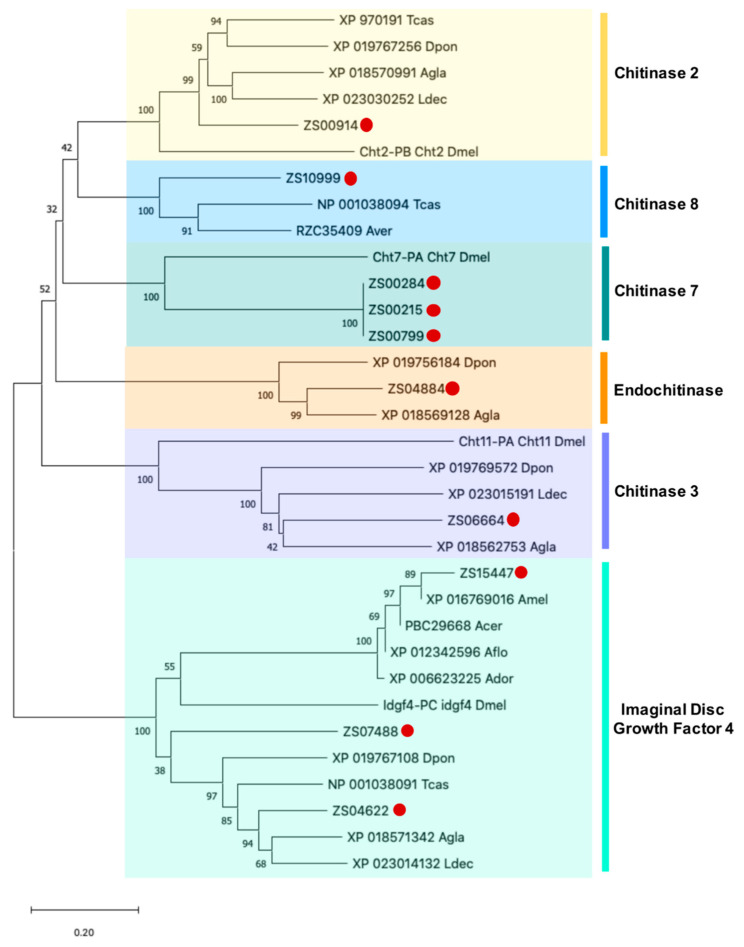
Phylogenetic analysis of putative IDGF4-chitinase proteins of *Z. subfasciatus* and other species. The phylogenetic tree was generated by MEGA X using the neighbor-joining method with 1000 bootstrap replicates. The accession number or symbol gene is shown. Amino acid sequences from other species used for comparison: Agla (*Anoplophora glabripennis*), Acer (*Apis cerana cerana*), Ador (*Apis dorsata*), Aflo (*Apis florea*), Amel (*Apis mellifera*), Aver (*Asbolus verrucosus*), Dpon (*Dendroctonus ponderosae*), Ldec (*Leptinotarsa decemlineata*), Tcas (*Tribolium castaneum*), Dmel (*Drosophila melanogaster*). The proteins were clustered into chitinase 2, chitinase 3, chitinase 7, chitinase 8, endochitinase and Imaginal Disc Growth Factor 4 groups. Colored boxes represent proteins belonging to the same group. Putative targets for PF2 lectin are identified by a red dot. The scale bar represents the branch lengths.

Based on this analysis, we found 10 candidates for IDGF4-chitinase proteins with a length range of 179 to 935 amino acids. According to the phylogenetic analysis, the putative IDGF4-chitinases are clustered with their homologs into six groups: chitinase 2 (1 unigene), chitinase 3 (1 unigene), chitinase 8 (1 unigene), endochitinase (1 unigene), chintinase 7 (3 unigenes) and Imaginal Disc Growth Factor 4 (3 unigenes). The last group of the unigenes ZS15447, ZS07488, and ZS04622 were closely related to chitinase-like proteins Idgf4 isoform X2 of *Apis mellifera* (western honeybee; XP_016769016), *D. ponderosae* (XP_019767108) and *A*. *glabripennis* (XP_018571342), respectively (supported by bootstrap values of 89%, 38% and 94%, respectively). These results support the presence of multiple IDGF4 genes in *Z. subfasciatus* (Table 3, Figure 5).

**Figure 6:**
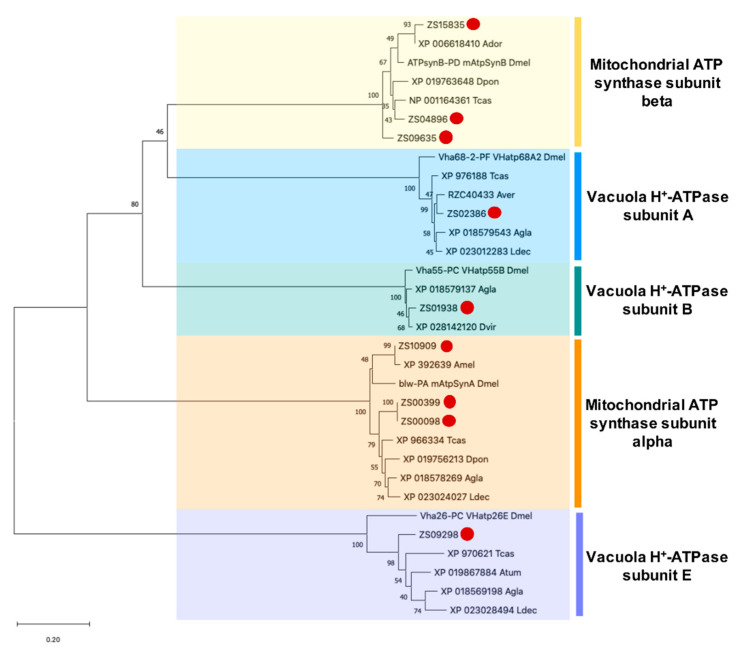
Phylogenetic analysis of putative ATP synthase subunit and V-type H+-ATPase proteins of *Z. subfasciatus* and other species. The phylogenetic tree was generated by MEGA X using the neighbor-joining method with 1000 bootstrap replicates. The accession number or symbol gene is shown. Amino acid sequences from other species used for comparison: Atum (*Aethina tumida*), Agla (*Anoplophora glabripennis*), Ador (*Apis dorsata*), Amel (*Apis mellifera*), Aver (*Asbolus verrucosus*), Dpon (*Dendroctonus ponderosae*), Dvir (*Diabrotica virgifera virgifera*), Ldec (*Leptinotarsa decemlineata*), Tcas (*Tribolium castaneum*), Dmel (*Drosophila melanogaster*). The proteins were clustered into mitochondrial ATP synthase subunits alpha and beta, and vacuola H+-ATPase subunit A, B and E groups. Colored boxes represent proteins belonging to the same group. Putative targets for PF2 lectin are identified by a red dot. The scale bar represents the branch lengths.

Nine unigene sequences with an average length of 2900 bp encoding for putative ATP synthase subunits or V-type H+-ATPase subunits with an average length of 417 amino acids were identified in the transcriptome. Of these, we obtained the complete ORF for five (Table 3). According to the phylogenetic analysis of the unigenes (with 43–100% of bootstrap support) and their homologs obtained by blast, six annotated as mitochondrial ATP synthase subunits alpha (3 unigenes) and beta (3 unigenes). In addition, of those classified as alpha, two sequences (ZS00399 and ZS00098) could be isoforms of the same gene. Another 3 unigenes were annotated and classified as V-type H+-ATPase subunit A (ZS02386), B (ZS01938), and E (ZS09298, Figure 6).

**Figure 7:**
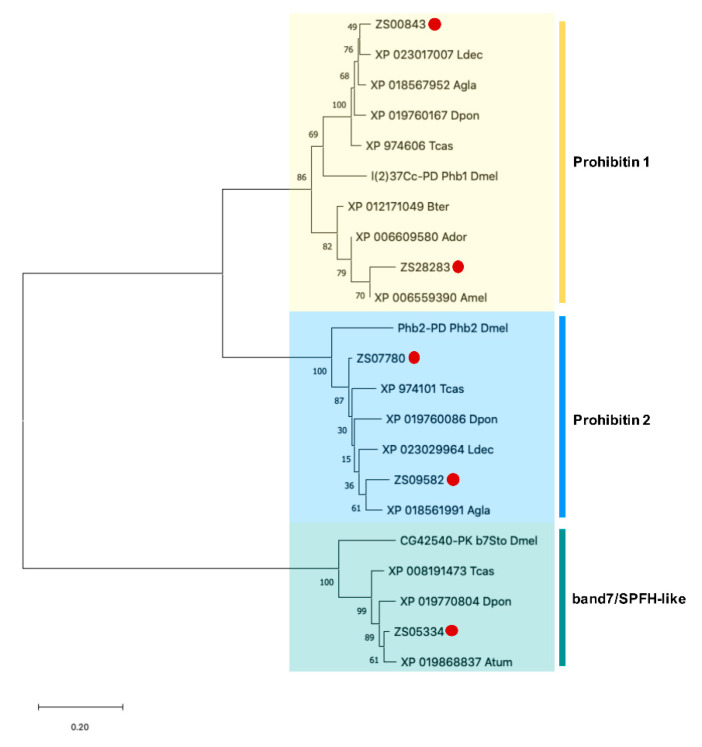
Phylogenetic analysis of putative prohibitin proteins of *Z. subfasciatus* and other species. The phylogenetic tree was generated by MEGA X using the neighbor-joining method with 1000 bootstrap replicates. The accession number or symbol gene is shown. Amino acid sequences from other species used for comparison: Atum (*Aethina tumida*), Agla (*Anoplophora glabripennis*), Ador (*Apis dorsata*), Amel (*Apis mellifera*), Bter (*Bombus terrestris*), Dpon (*Dendroctonus ponderosae*), Ldec (*Leptinotarsa decemlineata*), Tcas (*Tribolium castaneum*), Dmel (*Drosophila melanogaster*). The proteins were clustered into prohibitin 1, prohibitin 2 and band7/SPFH-like groups. Colored boxes represent proteins belonging to the same group. Putative targets for PF2 lectin are identified by a red dot. The scale bar represents the branch lengths.

We also identified five sequences as candidates encoding prohibitin proteins with an average length of 227 amino acids, for two of these, ZS00843 and ZS09582, we obtained the complete ORF with a length of 276 and 309 amino acids, respectively (Table 3). With a strong support the phylogenetic analysis showed that amino acid sequences encoded by these unigenes classified in the prohibitin 1 (2 unigenes), prohibitin 2 (2 unigenes), and band7/SPFH-like (1 unigene) families suggesting the presence of at least two different genes with the potential function of prohibitin 1 and two genes with the function of prohibitin 2 in *Z. subfasciatus* (Figure 7).

The search for potential PF2 lectin target sequences also allowed us to identify 3 unigenes encoding for mitochondrial-processing peptidase proteins (MPP) that were classified into subunit alpha (1 unigene) and beta (2 unigenes, ZS03356 and ZS04132), the latter could be isoforms of the same gene (Table 3, Figure S1). Finally, we found 2 unigenes encoding α-amylase proteins (ZS13222 and ZS18820) and 1 unigene encoding a mitochondrial NADH-ubiquinone oxidoreductase protein (ZS03487) (Table 3). The phylogenetic analysis of these sequences is shown in Figures S2 and S3, respectively. The sequences encoded by the unigenes ZS13222 and ZS18820 are related to A. mellifera α-amylase (AAM20738) and α-amylase 2-like A. dorsata (south and southeast Asian honeybee; XP_006614569), respectively. While that for mitochondrial NADH-ubiquinone oxidoreductase protein, ZS03487, is closely related to the mitochondrial NADH-ubiquinone oxidoreductase 75 kDa subunit of *A. glabripennis* (XP_018567499).

## 4. Discussion

The infestation of the stored common bean by *Z. subfasciatus* alters seed viability and nutritional value, and causes significant productivity and financial losses. Although important efforts have been made to search for strategies to control this pest, few studies have been conducted at the genomic level and little information on *Z. subfasciatus* is available from genomic and protein public databases. To our knowledge, there are only 40 nucleotide sequences and 26 amino acid sequences stored in the NCBI database. In the present study, we report the first transcriptomic analysis of *Z. subfasciatus* larvae that contains 29,029 unigenes with an average length of 1168 bp; we were able to assign a functional annotation for 18,032 (62.1%) assembled unigenes using public databases. Our data show that *Z. subfasciatus* shares greater similarity with *A. glabripennis* (63.5%) than with *T. castaneum* (8.3%) indicating that it might be phylogenetically closer to *A. glabripennis*. In agreement with this, a comparison with four species from the order Coleoptera showed that *Z. subfasciatus* has the highest number of putative orthologs with *A. glabripennis*, followed by *L. decemlineata*, and then *T. castaneum* and *D. ponderosae*. On the other hand, it was not possible to identify a probable function for 10,997 (37.9%) sequences, some of which were greater than 2000 bp, suggesting that several could be new genes, unique proteins or non-coding RNA sequences of *Z. subfasciatus*. Furthermore, the functional annotation of the *Z. subfasciatus* larvae transcriptome allowed us to categorize and to determine the classification of the most represented proteins in Biological Processes, Molecular Functions, and Metabolic Pathways. About 4640 InterPro families, 66 biological processes, 39 molecular functions, and 12 metabolic pathways are represented in the *Zabrotes* larval transcriptome. When the most represented GO terms between the transcriptomes of *Z. subfasciatus* and those Coleopterous insects with the highest putative orthologs were compared marked similarities were found in metabolic, catabolic, and binding processes [21,25]. The most represented GO categories tend to be very similar in closely related organisms (within the same order). However, for the Cellular Component classification, the most represented category in *L. decemlineata* was protein complex [26], while intracellular stood out in the *Z. subfasciatus* transcriptome. Such variations may occur between transcriptomes from different developmental stages or may be a sign of transcriptome incompleteness. So far, this provides a dramatic increase in the genetic resources for *Z. subfasciatus* and will help to support studies on genetic expression of this insect, as well as studies focusing on the biology and physiology of closely related organisms. Furthermore, this information could serve to accelerate the development of pest management tools through the analysis of potential protein targets of insecticidal molecules.

The toxicity of synthetic insecticides has encouraged research into alternative pest control strategies. Several plant proteins such as, lectins, chitinases, arcelins, and glycohydrolases exert entomotoxic activity and offer the promise of environmentally safe options for pest control. The insecticidal mechanism of lectins relies on the specific recognition of glycans which can be part of proteins, forming glycoproteins. Therefore, although a protein is ubiquitous, its glycosylation pattern can vary between species. In insects, protein glycosylation profiles have been observed to change depending on the species and reproductive and developmental stages [27]. Our research group has shown that PF2 is highly toxic for the first larval stages of *Z. subfasciatus* [10]. Although we have identified several proteins and glycoproteins that could be the targets of PF2 lectin in the larval midgut, the mechanism(s) of action remain unclear. Thus, we are directing our current efforts towards clarifying the insecticidal processes of PF2. We have used information provided from our transcriptome database to identify 30 unigenes encoding for potential PF2 lectin targets. The translated sequences of several of these targets have putative glycosylation sites and could provide us with the building blocks to design strategies for elucidating the molecular mechanisms of the entomotoxic effect of PF2 on the bean weevil larvae.

IDGFs are proposed PF2 targets in *Z. subfasciatus*; they belong to a family of glycoproteins classified as class V chitinases. Unlike conventional chitinases class V chitinases have 24 additional amino acid residues and lack chitinase enzymatic activity. In insects, these proteins are important for regulating growth and proliferation [28,29]. IDGF4 genes are involved in the molting process and are essential for organizing the exoskeletal barrier in these organisms [30]. IDGFs studies generally focus on functional characterization in model Diptera and Lepidoptera insects, few report findings for non-model species such as the bruchid, *Z. subfasciatus*. In this work, we report the presence of multiple IDGF genes expressed in *Z. subfasciatus* larvae. These data agree with observations in other insects such as *T. castaneum*, with a total of 22 genes encoding chitinases and chitinase-like proteins (IDGFs) and *Drosophila* with 16 genes encoding 10 chitinases and six IDGF [29]. All deduced amino acid sequences identified in this work for *Z. subfasciatus* IDGFs exhibited one potential N-glycosylation site; this supports the idea that IDGFs could play a role as a target receptor for PF2 lectin.

Other putative receptors for PF2 in *Z. subfasciatus* are mitochondrial ATP synthase and Vacuolar-type ATPase. While ATP synthase is mainly associated with cell energy production, recent reports indicate that the beta subunit of mitochondrial ATP synthase is also located on the plasma membrane of insect cells where it acts as receptor of a circulating lipoprotein for the midgut and fat body cells of *Panstrongylus megistus*, probably mediating lipid transfer to the insect’s fat body [31]. Vacuolar-type ATPases belong to a family of ATP-dependent proton pumps relevant in various membrane trafficking pathways that also function to acidify the lumen of some organelles and cellular compartments. In insects, the V-ATPases are present on the midgut brush border, and by acidification of the intestinal lumen, they provide proton-driving force for the secondary active transport of nutrients across this membrane [32]. In the present work, nine unigene sequences of ATP synthase and Vacuolar H+-ATPase subunits were identified. The analysis of potential N-glycosylation of these proteins predicted two sites for ATP synthase subunit beta, and three for V-ATPase subunits alpha and beta. Studies carried out in *T. castaneum* glycoproteins revealed that V-type ATPases are N-glycosylated [33]. Plasma membrane V-ATPase isolated from the midgut and Malpighian tubules of *Manduca sexta* (tobacco hornworm) is a N-glycosylated protein [34], while V-ATPase subunits from *Acyrthosiphon pisum* (pea aphid), *D. melanogaster* (common fruit fly), *A. mellifera*, *Bombyx mori* (silkworm), and *T. castaneum* show interaction with *Galanthus nivalis* (snowdrop) lectin indicating the presence of carbohydrates. The predicted glycosylation of *Z. subfasciatus* ATP synthase and V-ATPase subunits spur our continued study of them as potential target proteins for PF2 lectin. PF2 interaction with the β-chain of ATP synthase could interfere with both the production of ATP and the accumulation of lipids, while we might expect the interaction of PF2 with V-ATPase alpha and beta subunits affect lumen acidification and active transport of nutrients in *Z. subfasciatus* larvae. Thus, these two proteins represent key candidates for explaining PF2 insecticidal activity.

We have previously reported that an α-amylase isoform from *Z. subfasciatus* larvae midgut interacts with PF2. Amylases are an important family of enzymes required for the survival of insect larvae due to their involvement in carbohydrate metabolism [35]. We found two unigenes encoding α-amylase proteins (ZS13222 and ZS18820). The deduced amino acid sequences for these unigenes each showed one potential N-glycosylation site. Interestingly, the sequences obtained are more closely related to A. mellifera than to other coleopterans and failed to get the best blastx hit to a *Z. subfasciatus* α-amylase sequence already available in the NCBI database. This suggests the existence of variants that differ from constitutive amylase. Future experiments will be necessary to explore the specific expression of amylases obtained in this work at different developmental stages of the insect.

The mitochondrial-processing peptidase and mitochondrial NADH-ubiquinone oxidoreductase could be other putative PF2 targets. Mitochondrial-processing peptidase catalyzes the cleavage of N-terminal signal sequences of precursor proteins targeted for transport from cytosol to the mitochondria including those internalized into the matrix [36,37]. We found three unigenes of mitochondrial-processing peptidase in *Z. subfasciatus* larvae. Mitochondrial NADH-ubiquinone oxidoreductase is an enzyme of the mitochondrial electron transport chain and plays an important role during insect development as tissues are restructured and obsolete cells are destroyed by programmed cell death during metamorphosis [38]. In lepidopterans, such as *Chilo partellus* (spotted stalk borer) and *M. sexta*, changes were observed in mitochondrial oxidative activities during the larval-pupal-adult transitions [38,39,40]. However, the deduced amino acid sequences of mitochondrial-processing peptidase and mitochondrial NADH-ubiquinone oxidoreductase we identified showed no potential N-glycosylation sites and no reports were found of glycosylation of both proteins in other organisms. This suggests that PF2 recognition of these proteins is not likely to occur through a protein–carbohydrate interaction.

Another interesting target for PF2 in *Z. subfasciatus* is prohibitin. Prohibitins (PHBs) are multifunctional, but their main roles are as receptors of various cellular compartments such as the nucleus, nucleolus, mitochondria, endoplasmic reticulum, and plasma membrane [41,42]. PHBs have been associated with multiple functions depending on their intercellular location. In insects, PHBs are thought vital for normal development, since PHB1 and PHB2 have been implicated in the regulation of cell proliferation and apoptosis [12]. PHB1 is a receptor for the Cry3Aa protein from *Bacillus thuringiensis* (Gram-positive, soil-dwelling bacterium), making it a target protein for pest insect control. The putative prohibitin proteins of *Z. subfasciatus* were identified with five unigenes, two of which were for PHB1 and three for PHB2. PHBs have been reported to undergo post-translational modifications including glycosylation [41,43]; we report putative glycosylation sites for both PHBs.

In general, the sequences grouping of the putative PF2 lectin targets show strong support (>70% bootstrap), but the phylogenetic relationships among some of them remain unresolved. This may be because in some cases it was not possible to assemble the complete transcript which resulted in incomplete protein sequences. Therefore, nodes with low bootstrap values could be the result of a deficient number of informative sites from the alignment [44]. To solve this in related future investigations it will be necessary to obtain the complete sequence of these genes, in the meantime the specific sequences of *Z. subfasciatus* obtained in the present work will allow designing probes for gene expression and gene silencing studies, which may help unveil the role of putative PF2 targets in weevil development and physiology.

## 5. Conclusions

In conclusion, we report the first transcriptome of *Z. subfasciatus* that includes 29,029 assembled unigenes from 92 million paired-end reads. The data generated in this work will provide an important source of genomic data and will serve as a reference in studies on the biology of *Z. subfasciatus*. Our analysis of the transcriptome allowed us to identify the most active processes at the transcriptional level in *Z. subfasciatus* larvae, as well as gene families that code for possible targets of the PF2 lectin. This will provide information that will increase our understanding on the *Z. subfasciatus* larval development and relevant processes, as well as aid us in developing studies to evaluate the mechanisms of PF2 toxicity in this insect.

## Figures and Tables

**Table 1:** Summary statistics of *Z. subfasciatus* transcriptome assembly.

Total NUMBER of Assembled Unigenes	29,029
Total bases pairs (Mbp)	33.9
Smallest unigene (bp)	200
Largest unigene (bp)	15,454
Mean length unigene (bp)	1168.9
Unigenes over 1 kbp	10,379
Unigenes over 10 kbp	23
Unigenes with open reading frames	21,380
N90	443
N70	1285
N50	2196
GC percentage (%)	39
Proportion of N bases (%)	0.05

**Table 2:** Top 30 InterPro domains distribution of the unigenes assembled.

InterPro Domains	Number of Unigenes
(IPR013087) Zinc finger Cys2His2-type	440
(IPR000719) Protein kinase domain	195
(IPR020846) Major facilitator superfamily domain	137
(IPR017986) beta-transducin-repeat-containing domain	135
(IPR000504) RNA recognition motif domain	132
(IPR001841) Zinc finger, RING (Really Interesting New Gene)-type	94
(IPR007110) Immunoglobulin-like domain	94
(IPR020683) Ankyrin repeat-containing domain	91
(IPR002048) EF-hand domain	80
(IPR013026) Tetratricopeptide repeat-containing domain	71
(IPR001254) Serine proteases, trypsin domain	71
(IPR014001) Helicase superfamily 1/2, ATP-binding domain	71
(IPR001650) Helicase, C-terminal	70
(IPR027806) Harbinger transposase-derived nuclease domain	67
(IPR005225) Small GTP-binding protein domain	62
(IPR001849) Pleckstrin homology domain	61
(IPR001478) PDZ domain	61
(IPR001452) SH3 domain	60
(IPR003961) Fibronectin type III	57
(IPR000742) EGF-like domain	55
(IPR000210) BTB/POZ domain	55
(IPR001356) Homeobox domain	54
(IPR003439) ABC transporter-like	52
(IPR000477) Reverse transcriptase domain	50
(IPR013098) Immunoglobulin I-set	50
(IPR002557) Chitin binding domain	50
(IPR011545) DEAD/DEAH box helicase domain	48
(IPR001245) Serine-threonine/tyrosine-protein kinase, catalytic domain	42
(IPR017452) GPCR, rhodopsin-like, 7TM	41
(IPR003959) ATPase, AAA-type, core	38

**Table 3:** Unigenes encoding to putative lectin PF2 targets of *Z. subfasciatus*.

Unigen ID	Length (pb)	Open Reading Frame (aa)	BLASTX Best Hit (Accession Number/Description)	Identity (%)	E-Value	Gly Sites ^a^	Full Length ^b^
**Alpha amylase**							
ZS18820	393	128	AAM20738.1 | AF259649_1alpha-amylase [*Apis mellifera mellifera*]	100.0	3.78^−62^	2	No
ZS13222	698	104	AAM20738.1 | AF259649_1alpha-amylase [*Apis mellifera mellifera*]	100.0	2.57^−105^	1	No
**IDGF4 (Imaginal Disc Growth Factor 4)**							
ZS15447	541	179	XP_016769016.1 | PREDICTED: chitinase-like protein Idgf4 isoform X2 [*Apis mellifera*]	100.0	1.08^−126^	1	No
ZS04622	2177	351	XP_018571342.1 | PREDICTED: chitinase-like protein Idgf4 isoform X2 [*Anoplophora glabripennis*]	92.7	0	1	No
ZS07488	1479	245	XP_018571343.1 | PREDICTED: chitinase-like protein Idgf4 [*Anoplophora glabripennis*]	89.9	4.63^−109^	1	No
**Chitinase**							
ZS00914	4653	426	XP_018570991.1 | PREDICTED: probable chitinase 2 [*Anoplophora glabripennis*]	81.9	0	-	No
ZS10999	928	196	XP_018570656.1 | PREDICTED: acidic mammalian chitinase-like [*Anoplophora glabripennis*]	78.1	2.53^−58^	-	No
ZS00284	6248	935	XP_018575205.1 | probable chitinase 10 [*Anoplophora glabripennis*]	94.5	0	-	No
ZS00215	6614	935	XP_018575205.1 | probable chitinase 10 [*Anoplophora glabripennis*]	94.5	0	-	No
ZS00799	4836	935	XP_018575205.1 | probable chitinase 10 [*Anoplophora glabripennis*]	94.5	0	-	No
ZS04884	2088	551	XP_018569128.1 | PREDICTED: endochitinase [*Anoplophora glabripennis*]	84.5	0	-	No
ZS06664	1647	479	XP_018562753.1 | PREDICTED: chitinase-3-like protein 2 [*Anoplophora glabripennis*]	74.7	0	-	No
**Mitochondrial-processing peptidase subunit**							
ZS06108	1764	545	XP_018566230.1 | PREDICTED: mitochondrial-processing peptidase subunit alpha [*Anoplophora glabripennis*]	87.5	0	2	Yes
ZS03356	2689	208	XP_018560943.1 | PREDICTED: mitochondrial-processing peptidase subunit beta [*Anoplophora glabripennis*]	95.2	1.40^−158^	-	No
ZS04132	2348	376	XP_018560943.1 | PREDICTED: mitochondrial-processing peptidase subunit beta [*Anoplophora glabripennis*]	95.2	1.19^−158^	-	No
**NADH-ubiquinone oxidoreductase subunit mictochondrial**							
ZS03487	2626	423	XP_018567499.1 | PREDICTED: NADH-ubiquinone oxidoreductase 75 kDa subunit, mitochondrial [*Anoplophora glabripennis*]	92.0	0	-	No
**Prohibitin 1**							
ZS28283	208	51	XP_006559390.1 | PREDICTED: protein l(2)37Cc [*Apis mellifera*]	100.0	1.00^−16^	-	No
ZS00843	4761	276	XP_018567952.1 | PREDICTED: protein l(2)37Cc [*Anoplophora glabripennis*]	98.9	1.70^−163^	1	Yes
**Prohibitin 2**							
ZS07780	1423	209	XP_974101.1 | PREDICTED: prohibitin-2 isoform X1 [*Tribolium castaneum*]	94.9	1.41^−133^	2	No
ZS09582	1115	309	AEE63210.1 | unknown [*Dendroctonus ponderosae*]	90.6	3.31^−180^	4	Yes
**Band7 / SPFH-like**							
ZS05334	1961	291	XP_019868837.1 | PREDICTED: band 7 protein AGAP004871 isoform X1 [*Aethina tumida*]	96.6	0	-	No
**Mitochondrial ATP synthase subunit alpha**							
ZS10909	937	296	XP_016919804.1 | PREDICTED: ATP synthase subunit alpha, mitochondrial [*Apis cerana*]	100.0	0	-	No
ZS00399	5790	556	XP_966334.1 | PREDICTED: ATP synthase subunit alpha, mitochondrial [*Tribolium castaneum*]	95.6	0	-	Yes
ZS00098	7710	556	XP_966334.1 | PREDICTED: ATP synthase subunit alpha, mitochondrial [*Tribolium castaneum*]	95.6	0	-	Yes
**Mitochondrial ATP synthase subunit beta**							
ZS15835	519	173	KOB78881.1 | ATP synthase subunit beta [*Operophtera brumata*]	97.7	1.25^−117^	-	No
ZS09635	1106	357	XP_018562273.1 | PREDICTED: ATP synthase subunit beta, mitochondrial [*Anoplophora glabripennis*]	97.4	0	-	No
ZS04896	2083	517	NP_001164361.1 | ATP synthase subunit beta, mitochondrial [*Tribolium castaneum*]	94.5	0	2	Yes
**Vacuola H^+^-ATPase subunit A**							
ZS02386	3225	614	XP_018579543.1 | PREDICTED: V-type proton ATPase catalytic subunit A [*Anoplophora glabripennis*]	98.7	0	3	Yes
**Vacuola H^+^-ATPase subunit B**							
ZS01938	3568	496	XP_018579137.1 | PREDICTED: V-type proton ATPase subunit B [*Anoplophora glabripennis*]	99.2	0	3	Yes
Vacuola H^+^-ATPase subunit E							
ZS09298	1162	189	XP_018569198.1 | PREDICTED: V-type proton ATPase subunit E [*Anoplophora glabripennis*]	84.1	4.25^−99^	-	No

^a^ Glycosylation sites. ^b^ Open Reading Frame complete.

## Supplementary Materials

The following are available online at https://www.mdpi.com/2075-4450/11/11/736/s1: Figure S1: Phylogenetic analysis of putative mitochondrial-processing peptidase (MPP) subunit proteins of *Z. subfasciatus* and other species, Figure S2: Phylogenetic analysis of putative α-amylase proteins of *Z. subfasciatus* and other species, Figure S3: Phylogenetic analysis of putative Mitochondrial NADH-ubiquinone oxidoreductase proteins of *Z. subfasciatus* and other species, Table S1: Summary of annotation statistics of assembled unigenes, File S1: List of GO-slim terms represented for the assembled *Z. Subfasciatus* transcriptome.
