# Supplementary material for: Gene Sequences of Potential Targets of Insecticidal PF2 Lectin Identified from the Larval De Novo Transcriptome of the Mexican Bean Weevil (Zabrotes Subfasciatus; Boheman 1833)
Source: Insects. 2020 Oct 27;11(11):736. doi: 10.3390/insects11110736 (PMC7693830; doi:10.3390/insects11110736)
Supplement: Supplementary file 1 [file insects-11-00736-s001.zip › Supplementary Figures and Table.docx]

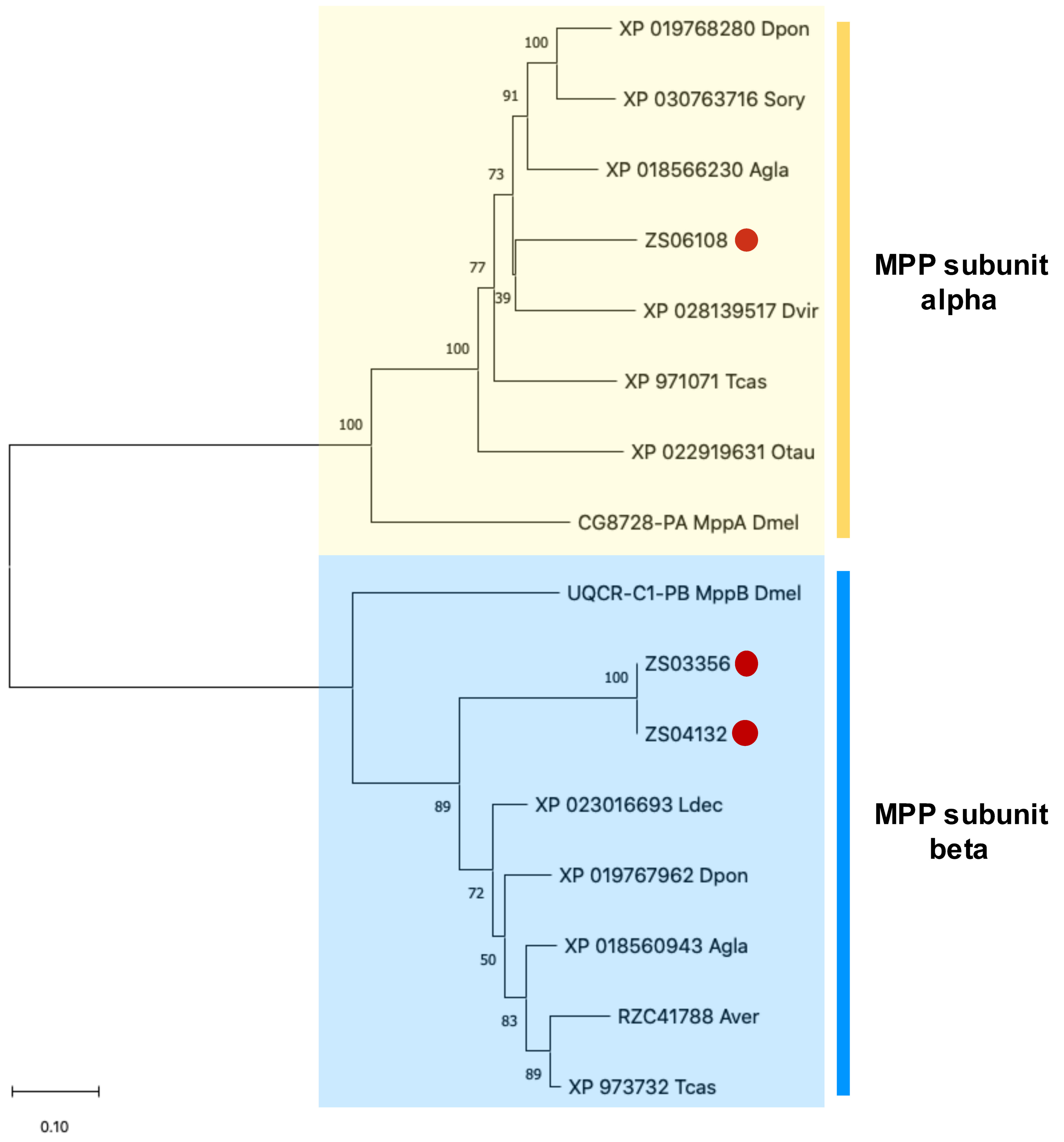


**Figure S1.** Phylogenetic analysis of putative mitochondrial-processing peptidase (MPP) subunit proteins of *Z. subfasciatus* and other species.

The phylogenetic tree was generated by MEGA X using the Neighbor-Joining method with 1,000 bootstrap replicates. The accession number or symbol gene is shown. Amino acid sequences from other species used for comparison: Agla (*Anoplophora glabripennis*), Aver (*Asbolus verrucosus*), Dpon (*Dendroctonus ponderosae*), Dvir (*Diabrotica virgifera virgifera*), Ldec (*Leptinotarsa decemlineata*), Otau (*Onthophagus taurus*), Sory (*Sitophilus oryzae*), Tcas (*Tribolium castaneum*), Dmel (*Drosophila melanogaster*). The proteins were clustered into mitochondrial-processing peptidase (MPP) subunit alpha and beta groups. Colored boxes represent proteins belonging to the same group. Putative targets for PF2 lectin are identified by a red dot. The scale bar represents the branch lengths.


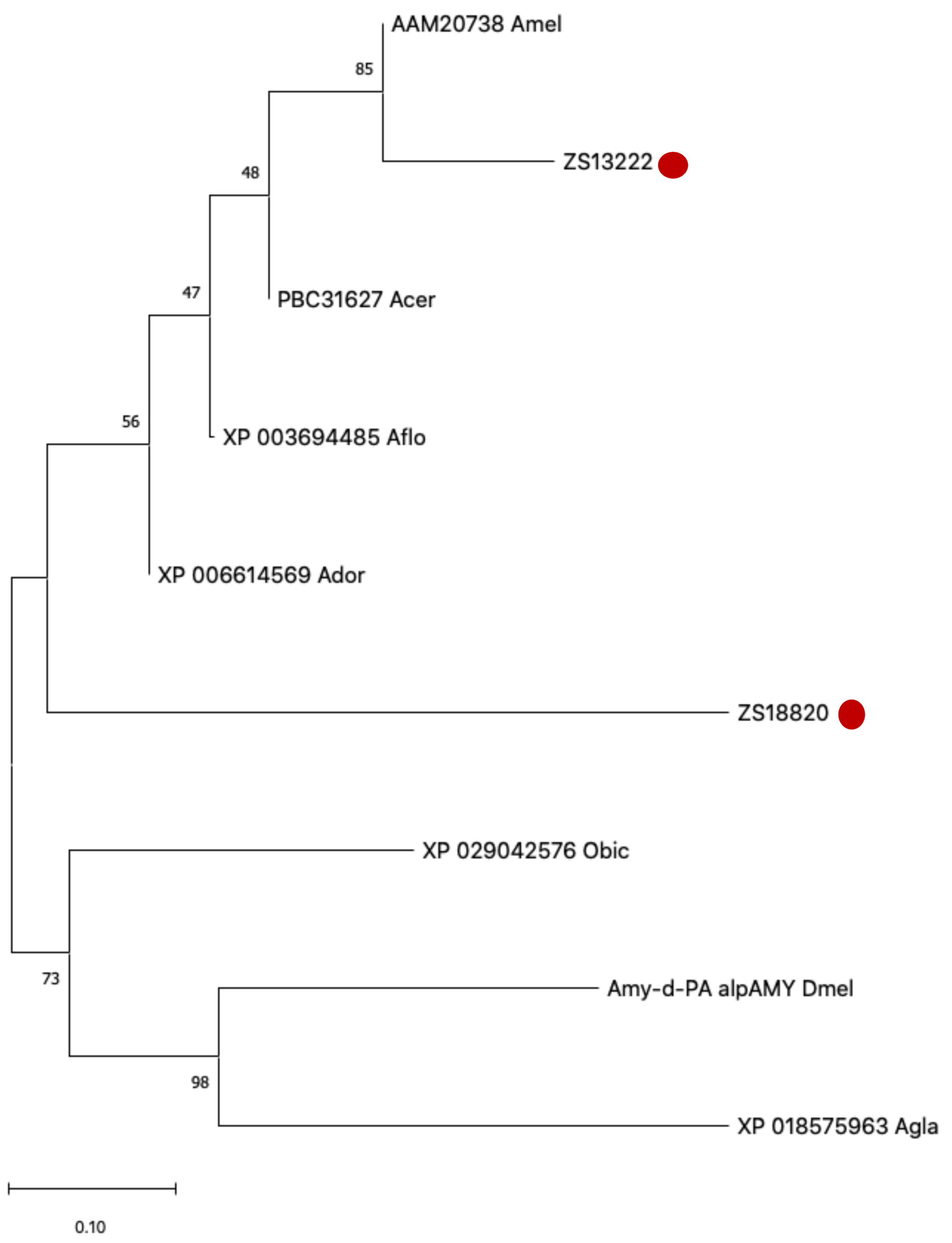


**Figure S2.** Phylogenetic analysis of putative α-amylase proteins of *Z. subfasciatus* and other species.

The phylogenetic tree was generated by MEGA X using the Neighbor-Joining method with 1,000 bootstrap replicates. The accession number or symbol gene is shown. Amino acid sequences from other species used for comparison: Agla (*Anoplophora glabripennis*), Acer (*Apis cerana cerana*), Ador (*Apis dorsata*), Aflo (*Apis florea*), Amel (*Apis mellifera*), Obic (*Osmia bicornis bicornis*), Dmel (*Drosophila melanogaster*). Putative targets for PF2 lectin are identified by a red dot. The scale bar represents the branch lengths.


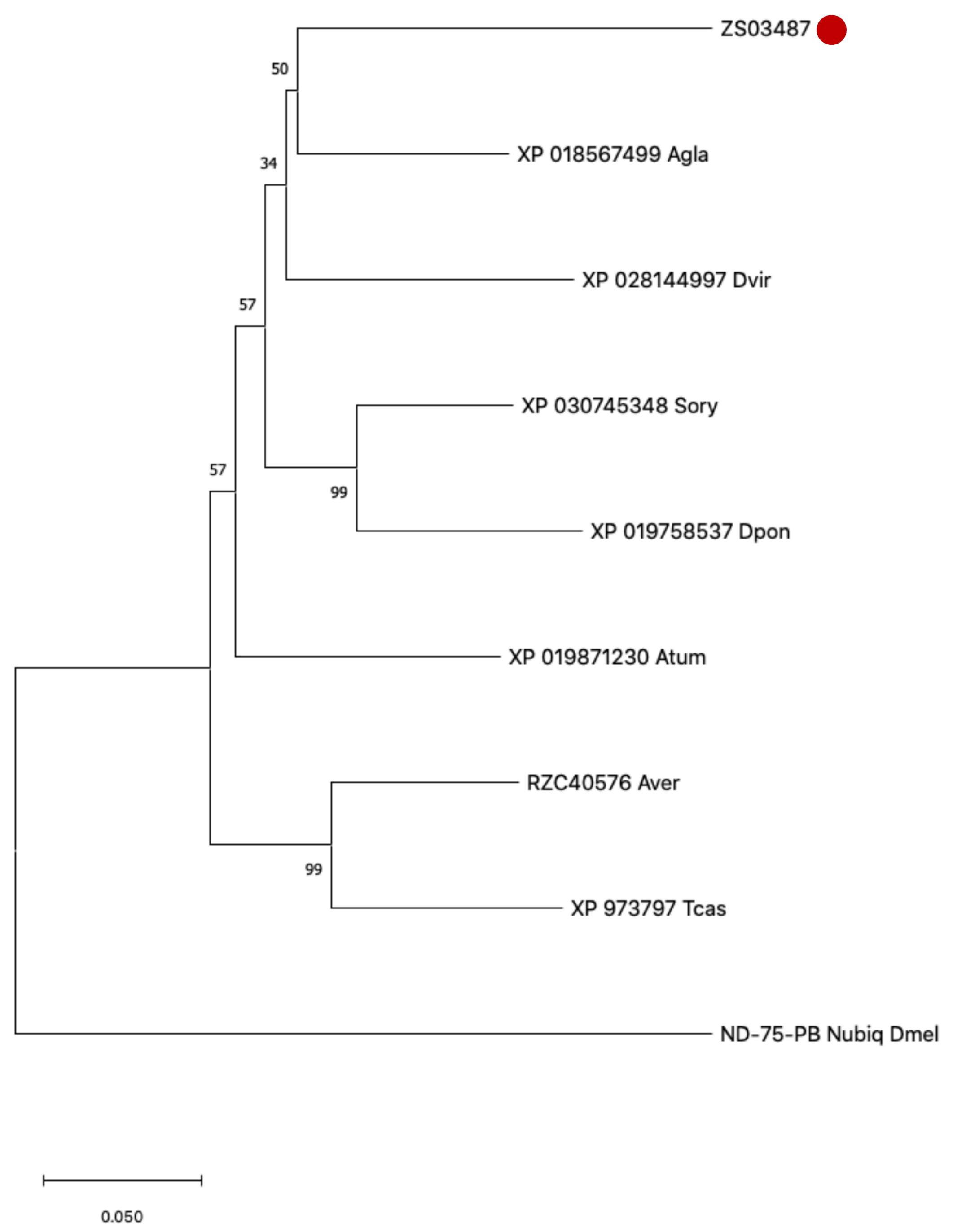


**Figure S3.** Phylogenetic analysis of putative Mitochondrial NADH-ubiquinone oxidoreductase proteins of *Z. subfasciatus* and other species.

The phylogenetic tree was generated by MEGA X using the Neighbor-Joining method with 1,000 bootstrap replicates. The accession number or symbol gene is shown. Amino acid sequences from other species used for comparison: Atum (*Aethina tumida*), Agla (*Anoplophora glabripennis*), Aver (*Asbolus verrucosus*), Dpon (*Dendroctonus ponderosae*), Dvir (*Diabrotica virgifera virgifera*), Sory (*Sitophilus oryzae*), Tcas (*Tribolium castaneum*), Dmel (*Drosophila melanogaster*). Putative target for PF2 lectin is identified by a red dot. The scale bar represents the branch lengths.

**Table S1.** Summary of annotation statistics of assembled unigenes.

| **Database** | **No. of Unigenes Annotated** | **Percentage (%)** |
| --- | --- | --- |
| RefSeq Non-redundant proteins | 15,079 | 51.9 |
| Non-redundant protein NCBI | 14,003 | 48.2 |
| UniProtKB/Swiss-Prot | 10,643 | 36.7 |
| InterPro | 15,830 | 54.5 |
| Gene Ontology | 11,741 | 40.4 |
| KEGG | 8,195 | 28.2 |
| *A. glabripennis* | 14,394 | 49.6 |
| *L. decemlineata* | 13,980 | 48.1 |
| *T. castaneum* | 13,714 | 47.2 |
| *D. ponderosae* | 13,184 | 45.4 |
| Total of unigenes annotated | 18,032 | 62.1 |
